# How does variation in the environment and individual cognition explain the existence of consistent behavioral differences?

**DOI:** 10.1002/ece3.451

**Published:** 2012-12-21

**Authors:** Petri T Niemelä, Anssi Vainikka, Jukka T Forsman, Olli J Loukola, Raine Kortet

**Affiliations:** 1 Department of Biology, University of OuluP.O. Box 3000, FI-90014, Finland; 2 Department of Biology, University of Eastern FinlandP.O. Box 111, FI-80101, Joensuu, Finland

**Keywords:** Animal personalities, behavioral flexibility, behavioral plasticity, behavioral syndromes, cognition, reaction norm

## Abstract

According to recent studies on animal personalities, the level of behavioral plasticity, which can be viewed as the slope of the behavioral reaction norm, varies among individuals, populations, and species. Still, it is conceptually unclear how the interaction between environmental variation and variation in animal cognition affect the evolution of behavioral plasticity and expression of animal personalities. Here, we (1) use literature to review how environmental variation and individual variation in cognition explain population and individual level expression of behavioral plasticity and (2) draw together empirically yet nontested, conceptual framework to clarify how these factors affect the evolution and expression of individually consistent behavior in nature. The framework is based on simple principles: first, information acquisition requires cognition that is inherently costly to build and maintain. Second, individual differences in animal cognition affect the differences in behavioral flexibility, i.e. the variance around the mean of the behavioral reaction norm, which defines plasticity. Third, along the lines of the evolution of cognition, we predict that environments with moderate variation favor behavioral flexibility. This occurs since in those environments costs of cognition are covered by being able to recognize and use information effectively. Similarly, nonflexible, stereotypic behaviors may be favored in environments that are either invariable or highly variable, since in those environments cognition does not give any benefits to cover the costs or cognition is not able to keep up with environmental change, respectively. If behavioral plasticity develops in response to increasing environmental variability, plasticity should dominate in environments that are moderately variable, and expression of animal personalities and behavioral syndromes may differ between environments. We give suggestions how to test our hypothesis and propose improvements to current behavioral testing protocols in the field of animal personality.

## Introduction

**Figure 1:**
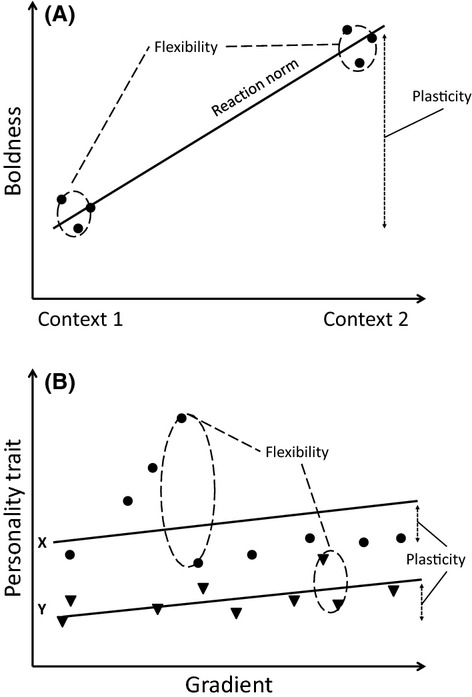
(A) Personality trait (here boldness) measured from the same individual in two different contexts: context 1 = feeding context and context 2 = mating context. Boldness has been measured three times in each contexts (black dots), from which one can see behavioral flexibility (dashed line area). Behavioral plasticity between contexts can be seen from the slope of the behavioral reaction norm (dashed arrows). In figure (B), boldness is measured multiple times from two individuals (X and Y, black dots and triangles, respectively) across environmental gradient in one context. Individual X shows higher behavioral flexibility (larger dashed line area) but similar plasticity (dashed arrows) compared with individual Y. Because individual X has higher flexibility, its behavioral plasticity could be considerably higher across some other environmental gradient, where this kind of plasticity would be adaptive. However, individual Y would still show similar, restricted plasticity, because of its limited potential for flexibility. Reaction norm from individual Y can be estimated more precisely, since its flexibility is considerably lower. In general, personality exists in cases where flexibility does not exceed consistency (repeatability) so that a behavioral reaction norm can be estimated for any given individual.

While the concept of animal personality (i.e. consistent between-individual differences in time and between contexts) has significantly improved our understanding about the evolution of individually consistent behavior (Kortet et al. 2010; Sih et al. 2012), it has simultaneously created a need to understand the plasticity of behavior (Dingemanse et al. 2010, 2012a; Mathot et al. 2012). In animal personality literature, behavioral plasticity is thought to be somewhat limited so that the amount of plasticity depends mostly on the individual (Dingemanse et al. 2010). Behavioral plasticity and animal personalities are best described by using the technical definition of personality through the concept of behavioral reaction norm (Smiseth et al. 2008; Dingemanse et al. 2010). A behavioral reaction norm defines how the average level of any given personality trait (such as boldness or aggressiveness) can differ in time or across an ecological gradient, between contexts and between individuals (Dingemanse et al. 2010) (Fig. 1). Therefore, behavioral plasticity is closely related to individual consistency of behavior: individuals expressing high consistency cannot be flexible (Fig. 1). Here, we define behavioral plasticity as the individual's average ability to respond to environmental stimulus across gradient (i.e. nonhorizontal reaction norm) (Fig. 1). Therefore, behavioral plasticity represents strictly the Genes × Environment interaction in the present paper. Within a context or a situation animal shows also behavioral flexibility (range of individual behaviors from which reaction norm is build) which can also depend on the slope of the behavioral reaction norm (Fig. 1). Behavioral flexibility and the slope of the reaction norm (plasticity) may be related to each other so that the more potential for flexibility individual has (greater variance around the reaction norm), the higher is the potential for plasticity (Fig. 1B). This is a valid assumption, since, for example, in predation threat level gradient nonflexible individuals show consistent behavior (horizontal reaction norm) while flexible individual is able to change behavior according to the threat and at the same time show nonhorizontal reaction norm.

The amount of behavioral plasticity an individual shows depends evolutionarily on the environment because its complexity and variability determine the realm individuals meet and have to react (Bonte et al. 2007; Mathot et al. 2012). Acquiring, processing, and implementing information entails costs (Greeno et al. 1996; Sternberg and Grigorenko 1997; Pravosudov et al. 2006; Overli et al. 2007; Coppens et al. 2010; Koolhaas et al. 2010) and may thus create a selection for different behavioral flexibility relative to environmental characteristics within a given range of information processing abilities (Sol et al. 2005). However, no unified theory exists about how consistent behavioral differences and amount of behavioral plasticity should relate to environmental variability and variation in costly animal cognition in the context of animal personality research.

Here, we use existing literature to review how environmental variation and individual differences in cognition explain the expression of behavioral flexibility and integrate it in a novel way to animal personality context. Thus, we create a conceptual framework that explains why some populations express consistent behavioral differences (i.e. personalities with low level of plasticity), while others do not or show weaker consistency than others. We will use a term “stereotypic behavioral type” for behavioral types that display limited behavioral flexibility independently of the environment, and term “responsive behavioral type” for behavioral types that can change their immediate behavior according to variation in environment. Highly flexible individuals (here, responsive) are able to respond to the stimulus optimally or near optimally and according to ecological context or situation and may thus have steep reaction norms (high plasticity) compared with nonflexible individuals (here, stereotypic), which show limited behavioral response to stimulus despite the context (relatively flat reaction norm, low plasticity).

## Evolution of animal cognition

Individuals need sensory mechanisms and neural processing abilities, i.e. cognition, to extract information from noisy and variable environmental cues to be used in important fitness decisions (Godfrey-Smith 2002; Dukas 2004). Here, we use a broad definition of cognition, and define it as individual's overall ability to acquire, retain, process, and use information. Cognitive learning is a common phenomenon in nature shared by species from invertebrates to mammals (Papini 2002). One of the most important suggested driving forces for the evolution of cognition is the variability of the environment (Bergman and Feldman 1995; Richerson and Boyd 2000; Godfrey-Smith 2002). In variable environments, cognition gives individuals ability to respond behaviorally to environmental fluctuation. Therefore, environments with greater variability favor cognition compared with environments with less variability (Bergman and Feldman 1995; Richerson and Boyd 2000; Godfrey-Smith 2002; Mery and Kawecki 2002; Kerr and Feldman 2003), as long as environmental cues are reliable (Kerr and Feldman 2003; McElreath and Strimling 2006). When environmental variability is high or unpredictable enough to prevent fitness increase by improving cognition, selection should disfavor enhanced cognition (Bergman and Feldman 1995; Kerr and Feldman 2003, but see Kerr and Feldman 2003). As environmental characteristics between populations may express great amount of spatiotemporal variation (Ruokolainen et al. 2009; Bezault et al. 2011; García-Carreras and Reuman 2011), different potential selection pressures from low to high for cognition is expected among populations.

Given that cognitive abilities vary between individuals, populations, and species (Sternberg and Grigorenko 1997; Healy and Braithwaite 2000; Wolf et al. 2008), some individuals are likely able to acquire and use information from variable environments more efficiently than others (e.g. Sol et al. 2005). Therefore, differences in animal cognition may affect the differences in behavioral flexibility (Sol et al. 2005). There are also, some indirect neurobiological and direct behavioral and genetic evidence about individual level differences within species about cognitive abilities (Feldker et al. 2003; Dukas 2004; Amy et al. 2012).

## Costs and benefits of cognition

Individuals with high abilities to process and extract information from the environment are capable of producing precise behavioral responses to environmental cues and thus, achieve higher fitness compared with individual with less efficient information processing abilities (Dukas and Bernays 2000; Dukas 2004). Dukas and Bernays (2000) for example, found that Grasshoppers, which were better learners, had higher growth rates giving them fitness advantage over nonlearners. However, since not all individuals or species express optimal responses to environmental stimuli, cognition must also include some costs (Dukas 1999). Cognition and memory is inherently costly to build and maintain, as brain and other nerve tissues and physiology needed for cognition and memory are energy demanding (Armstrong 1983; Laughlin et al. 1998; Purdon and Rapoport 1998; Dukas 1999; Isler and Van Schaik 2009a,b). Also, information acquisition from the environment to be used in cognitive behavioral decisions may induce time, reliability and predation costs (Sih 1992; Dewitt et al. 1998), which may select against cognition and therefore, against behavioral flexibility. There may also be evolutionary costs between cognition and some other, fitness related trait (Kawecki 2010). One proposed evolutionary cost is a functional trade-off between learning ability and some other aspect of performance or behavioral trait which prevent learning to evolve, even beneficial, since costs for that are higher than benefits (Kawecki 2010). Good example for this kind of evolutionary cost is reduced lifespan and reduced larval competitive ability in flies, *Drosophila melanogaster*, with high information processing abilities (Mery and Kawecki 2003; Kawecki 2010). In the great tit, *Parus major*, individuals with better problem solving ability produce larger clutches than nonsolvers but this effect does not result in higher number of offspring because individuals with better learning ability are more sensitive to disturbance and desert their nests more often (Cole et al. 2012). Thus, physio-ecological aspects can define the value of cognitive ability and behavioral flexibility according to environment.

## Empirical evidence for environment variation-dependent cognition and behavioral flexibility

There is evidence from closely related species that different variability of the environment may lead to corresponding variation in cognitive abilities and flexibility of behavior (e.g. Richerson and Boyd 2000). Norwegian rats (*Rattus norvegicus*), inhabiting variable environments are variable in their behavioral responses by acquiring constantly new food sources by using social cues. In contrast, black rats (*Rattus rattus*) inhabiting stable, nonvariable environments seem to express relatively fixed behaviors after juvenile learning period (e.g. Richerson and Boyd 2000). Same logic can be used to compare different populations within species. Clayton and Krebs (1994) showed that environmental stimulation confronted by an individual affected the brain structures needed for spatial computation within species. Also, these brain structures can change seasonally according to the use of complex spatial memory (Smulders et al. 1995; Clayton 1997). The environment experienced during ontogeny may also affect the cognitive abilities (Carere and Locurto 2011). The potential fitness value of information use likely depends also on the fitness importance of an ecological phenomenon (Stephens 1989), how information is used (Stephens 1989; Koops 2004; Seppänen et al. 2007), information reliability (i.e. noise level) (Koops 2004; McElreath and Strimling 2006; McLinn and Stephens 2006), and the qualities of an individual facing the environment.

Behavioral types likely have a basis on hormonal or neurotransmitter differences between individuals (e.g. Coppens et al. 2010), so that different behavioral types are based on different physiological qualities but also so that responsiveness requires high capacity for cognition. Also serotonin and dopamine levels may affect the level of behavioral flexibility (Coppens et al. 2010), implying that some individuals may have more direct stimulus – response chain to environmental cues compared with others leading some to being guided by the environment while others being guided by routines (Coppens et al. 2010 and references therein). Species using complex spatiotemporal information have different brain structure compared with species using simpler information (Sol et al. 2005; Pravosudov et al. 2006; Ward et al. 2012). Bird species with large brains are found to have higher survival in novel environments compared with birds with smaller brains (Sol et al. 2005). Also, environmental characteristics may define population level differences in brain structures (Healy and Braithwaite 2000; Sol et al. 2005; Gonda et al. 2012), and therefore affect cognitive abilities.

**Figure 2:**
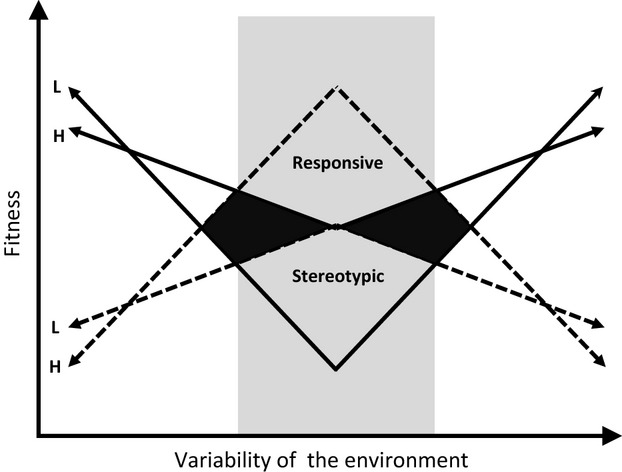
Behavioral type-dependent fitness benefits of information usage within and between behavioral types in environments with different environmental variability. In grey area the benefits of cognition exceeds its costs. Therefore, responsive behavioral types (dashed line) with high (H) and low (L) cognitive abilities dominate in these kinds of environments, compared with stereotypic behavioral types (solid lines) that instead dominate in invariable or highly variable environments (i.e. outside grey area). Variation in cognitive abilities within and between behavioral types leads to environment-dependent coexistence of different behavioral types (black area). In the grey area the high plasticity and flexibility in behavior potentially restricts the consistency in behaviors in time and across contexts and therefore, may limit the abundance or affect the expression of animal personalities.

Based on abovementioned literature, we conclude that if environment is spatially and/or temporally invariable or overly variable (in relation to the available information processing capacity), stereotypic behavioral types may dominate over responsive behavioral types because in such environment the maintenance costs of cognitive system of responsive individuals exceed the benefits. That is because in invariable or mechanistically repeated environment, adaptive responses to a certain stimuli are invariable and do not require behavioral flexibility brought by efficient cognition (Fig. 2). In moderately variable environments responsive behavioral types may have an advantage, since costs of cognition are covered by being able to recognize and use information despite of surrounding noise. Genetic between-individual variation in cognitive capacity can induce variance within and between responsive and stereotypic behavioral types (i.e. high and low cognitive ability in Fig. 2), and form a two dimensional space where both, responsive and stereotypic behavioral types can coexist (Fig. 2).

## Hypothesis: from behavioral plasticity to animal personalities

Our integrative framework is based on above reviewed literature on costly animal cognition. We use it to explain behavioral plasticity (in animal personality context) by clarifying the idea that individual cognition and environmental variability explains variation in behavioral flexibility (e.g. Clayton and Krebs 1994; Smulders et al. 1995; Clayton 1997; Richerson and Boyd 2000). Personality exists in cases where flexibility does not exceed consistency (repeatability) so that a behavioral reaction norm can be estimated for any given individual. As responsive behavioral types are more flexible, moderately variable environments should favor more behavioral flexibility and plasticity than invariable or highly variable environments, and thus finding statistically consistent behaviors (traditionally the same as animal personality) in those environments could be more difficult when compared with environments which favor high individual consistency in behavior (Fig. 2). Overall potential for flexibility in responsive individuals is maintained by enhanced cognition and depends on environmental variation (Bergman and Feldman 1995; Richerson and Boyd 2000; Godfrey-Smith 2002; Mery and Kawecki 2002; Feldker et al. 2003; Kerr and Feldman 2003; Dukas 2004; Sol et al. 2005; Amy et al. 2012), but behavioral plasticity, within the limits of flexibility, is an adaptive response to any environmental gradient in which plasticity is beneficial. Amount of behavioral flexibility defines the upper limit to behavioral plasticity, which could be expressed in any direction (i.e. positive or negative slope of reaction norm) depending on the amount and direction of plasticity environment favors. However, plasticity is not necessarily always beneficial, since in some cases, it would be more adaptive for an individual to express strictly consistent behavior. In these cases, also responsive individuals may express limited flexibility and potential for plasticity may therefore remain hidden (Fig. 1B). Therefore, we highlight the importance of assessing animal personality across wide environmental gradients and by using behavioral reaction norm-based approaches (Dingemanse et al. 2010). This way, we would be able to reveal true flexibility and plasticity and assess how they are related. When behavioral flexibility is too high, behavior may not be anymore described by any consistent, linear, or nonlinear, behavioral reaction norm. However, the reaction norm can adopt any form (Dingemanse et al. 2010), and variation around the reaction norm can be estimated to quantify the existing variance in behavioral flexibility: a dimension of animal personality that is traditionally not taken into account when defining the personality concept.

Behavioral flexibility and plasticity has implications also for behavioral syndromes among two or more personality traits. The consistent within- and between-individual associations between several behaviors (Dingemanse et al. 2012b; Garamszegi et al. 2012), for example between aggression and boldness, may not be tight and consistent among responsive behavioral types in all environments, contexts or situations, since different behaviors can be optimized individually according to context or situation by individuals with high cognitive ability and the association between behaviors may differ accordingly. If different behavioral types (i.e. responsive or stereotypic) are favored in environments with different variability, the existence and expression of animal personalities may depend on the variability of the environment.

## Testing the hypothesis

Idea that behavioral plasticity or expression of personalities depend on the environmental characteristics and their selective pressures on cognition can be tested, for example, by using protocols from animal cognition and memory research (Clayton and Krebs 1994; Smulders et al. 1995; Clayton 1997; Dukas and Bernays 2000; Mery and Kawecki 2002, 2003, Kawecki 2010). One can, for example, do comparative tests to examine individual differences in learning ability and expand the experiment to test, using animal personality protocols, if the expression of behavioral plasticity (slope of the reaction norm) depends on individual's overall learning capacity. To further test if behavioral flexibility is directly related to behavioral plasticity a large number of repeated tests should be conducted along different environmental qualities. We predict that the behavioral plasticity is favored if behaviorally flexible responses occurring consistently toward a certain direction in a certain environment give a fitness advantage in that environment. Thereby, behavioral plasticity could evolve as consistent (between different environments) selection against nonadaptive flexible behavioral reactions.

Another approach would be to use selection experiments. Selection lines can be easily produced in invertebrates as they produce several generations in a short time. For example, crickets have been used previously in animal personality and behavioral syndrome studies (Kortet and Hedrick 2007; Niemelä et al. 2012a,b,c). Also fruit flies have been used in cognition studies (Mery and Kawecki 2002, Kawecki 2010). To test our hypothesis, selection lines would be needed to be grown in environments with controlled amount of variability for at least two to three generations. The agent of variability could be, for example, food source, which would be changed spatially in controlled time intervals. After several generations, personality and behavioral plasticity experiments could be carried out to examine, if there are differences between the selection lines in the learning capacity, flexibility, and behavioral plasticity according to selective environmental gradient.

To gain support to our hypothesis, by using abovementioned methods, the amount of behavioral flexibility and environmental variation should relate the amount of behavioral plasticity or the expression of plasticity according to the amount of environmental variation (Figs 2), but any counter-evidence would falsify the framework for the tested system. However, learning itself could cause consistent behavior, and therefore strictly observational data cannot falsify our hypothesis.

Overall, behavioral assays used in animal personality studies should be designed so that individuals could not be able to learn in those situations. This is because, when repeated behavioral assays are conducted, animals may learn how to behave in the assay, and through learning behave consistently in the following assays. Therefore, efficient cognition does not always increase variance in behavior but may also lead to false detections of repeatable behaviors that are then erroneously concluded to indicate the existence of personalities or behavioral syndromes. Behavioral assays, like, for example, boldness tests could be conducted always in different, novel, environments to prevent the learning effect. Repeated novel but ecologically equal challenges could also give improved insights about individual differences in behavioral flexibility and plasticity compared with standard approaches neglecting the importance of learning and intrinsic differences in behavioral flexibility. This issue is of particular importance in natural systems where every individual has some experience about the environment and the current behavior is affected by learning. Therefore, to understand the evolutionary mechanisms, naïve individuals should be used in experimental assays examining the interplay between cognition and individually consistent behavior.

## Conclusion

Here, we have shortly reviewed literature on animal cognition to create a hypothesis on how the existence or expression of behavioral plasticity may depend on the interaction between environmental and individual characteristics. We have also introduced some ideas to test our hypothesis. We pinpoint that differences in behavioral plasticity arise from (1) differences in costly cognition; and (2) the level of variability of the environment. According to our reasoning, responsive behavioral types have superior cognitive ability (i.e. ability for behavioral flexibility) and thus greater ability to acclimatize quickly to moderately variable environments compared with stereotypic behavioral types. In invariable and highly variable environments, however, the cognitive ability of responsive behavioral types turns into a cost because physiological costs are paid without information gain and stereotypic behavioral types are favored. Therefore, our framework concludes that consistent behavioral differences are most likely limited to environments, which favor limited behavioral plasticity.
